# Exploring the Potential of Icelandic Seaweeds Extracts Produced by Aqueous Pulsed Electric Fields-Assisted Extraction for Cosmetic Applications

**DOI:** 10.3390/md19120662

**Published:** 2021-11-25

**Authors:** Natalia Castejón, Kristin Anna Thorarinsdottir, Ragnhildur Einarsdóttir, Kristberg Kristbergsson, Gudrún Marteinsdóttir

**Affiliations:** 1 School of Health Sciences, Faculty of Food Science and Nutrition, University of Iceland, 101 Reykjavik, Iceland; natalia.castejon@univie.ac.at (N.C.); rae22@hi.is (R.E.); kk@hi.is (K.K.); 2 TARAMAR Ehf., Miðnestorg 3, 245 Sandgerði, Iceland; kristin.anna@outlook.com; 3 Faculty of Life and Environmental Sciences, University of Iceland, 101 Reykjavik, Iceland

**Keywords:** macroalgae, *Ulva lactuca*, *Alaria esculenta*, *Palmaria palmata*, PEF-assisted extraction, bioactive compounds, green extraction, natural ingredients, cosmeceuticals

## Abstract

A growing concern for overall health is driving a global market of natural ingredients not only in the food industry but also in the cosmetic field. In this study, a screening on potential cosmetic applications of aqueous extracts from three Icelandic seaweeds produced by pulsed electric fields (PEF) was performed. Produced extracts by PEF from *Ulva lactuca*, *Alaria esculenta* and *Palmaria palmata* were compared with the traditional hot water extraction in terms of polyphenol, flavonoid and carbohydrate content. Moreover, antioxidant properties and enzymatic inhibitory activities were evaluated by using in vitro assays. PEF exhibited similar results to the traditional method, showing several advantages such as its non-thermal nature and shorter extraction time. Amongst the three Icelandic species, *Alaria esculenta* showed the highest content of phenolic (mean value 8869.7 µg GAE/g dw) and flavonoid (mean value 12,098.7 µg QE/g dw) compounds, also exhibiting the highest antioxidant capacities. Moreover, *Alaria esculenta* extracts exhibited excellent anti-enzymatic activities (76.9, 72.8, 93.0 and 100% for collagenase, elastase, tyrosinase and hyaluronidase, respectively) for their use in skin whitening and anti-aging products. Thus, our preliminary study suggests that Icelandic *Alaria esculenta*-based extracts produced by PEF could be used as potential ingredients for natural cosmetic and cosmeceutical formulations.

## 1. Introduction

In recent years, the demand for new bioactive compounds with potential health benefits has undergone a substantial increase. Many research groups have placed emphasis upon research on marine organisms, such as macroalgae, to find novel and sustainable sources of natural compounds for applications in the agri-food industry, pharmacology, foods and, more recently, in the field of cosmetics [1,2]. Macroalgae are a large and heterogeneous group of photosynthetic organisms characterized by a huge biodiversity and complex biochemical composition. According to their chemical structure and pigment content, macroalgae can be divided into three lineages including brown algae (*Phaeophyceae*), red algae (*Rhodophyta*) and green algae (*Viridiplantae*). Algal compounds are stored inside the cell cytoplasm or bound to cell membranes; thus, cell disruption is crucial for the valorization of algal biomass. Additionally, the cell wall composition is highly variable between algae species ranging from tiny membranes to multi-layered complex structures, making the recovery of algal products a challenge [3]. In general, seaweeds are excellent sources of polysaccharides, proteins, lipids, and a wide variety of secondary metabolites such us phenolic compounds, terpenoids, carotenoids, pigments and nitrogen derivatives [4,5,6]. Although primary metabolites have crucial importance, recent data have shown that the content of secondary metabolites determines the biological activities of seaweed extracts [7]. 

A growing concern for overall health and wellness, as well as awareness of harmful chemicals in everyday products, is driving to a global market of natural and organic ingredients [8]. Over the past years, consumer consciousness towards preference of natural ingredients and eco-friendly products has extended from the food industry to the cosmetic and personal care industry [9]. Furthermore, in the current context of global warming and ecological issues, there has been increasing public awareness of environmental issues. In light of these current concerns, consumers have turned their interests towards green, healthy and chemical-free products. As a result, the cosmetic industry is currently replacing toxic chemicals and harmful ingredients with novel and natural high-value compounds to produce “chemically-clean” beauty products [10]. 

Cosmetics have traditionally been defined as products to be applied to human body for cleansing, beautifying, or promoting attractiveness without affecting body structure or functions. However, new trends and recent consumer demands have promoted the development of novel products that supply multiple benefits with minimal efforts. The term cosmeceutical is now frequently used to describe cosmetic products with bioactive ingredients claiming to have medical or drug-like benefits [11]. Cosmeceuticals usually contain functional ingredients such as vitamins, phytochemicals, enzymes, antioxidants and/or essential oils [12]. Since a wide range of these bioactive compounds have been found in macroalgae, the investigation of new seaweeds and marine algae-derived extracts have proven to be a promising area of cosmeceutical and cosmetics studies [13,14]. 

A number of secondary metabolites derived from seaweeds are known for their valuable health beneficial effects on skin, such as photo-protective, moisturizing, antioxidant, anti-inflammatory and regenerative properties [15]. Based on these beneficial effects, algae are incorporated in cosmeceutical products such as sunscreen, anti-aging products, as well as for prevention of hyperpigmentation, while polysaccharides are used for keeping the skin moisturized and to prevent dryness [16]. During aging, the extracellular matrix proteins are susceptible to excessive activity of proteolytic enzymes such as collagenases and elastases, resulting in visible changes in the skin, such as wrinkles or the loss of skin elasticity. A promising approach to prevent extrinsic skin aging is the inhibition of collagenase and elastase activities by natural compounds. Plant extracts have been widely investigated and found to possess anti-collagenase and anti-elastase activities [17]. However, there is little information on the inhibitory enzymatic activities of seaweeds extracts. 

The most frequently applied extraction methods for the isolation of bioactives from seaweeds are based on conventional techniques. Nevertheless, the utilization of traditional methods has several drawbacks, such as the use of high volumes of organic solvents, longer extraction times, high temperatures, selectivity problems, high energy requirements, and coextraction of untargeted or interfering compounds [18]. Hence, new extraction techniques based on green chemistry principles have a potential interest [19]. 

Pulsed electric field (PEF) is an emerging, nonthermal and energy-efficient food processing technology [20]. PEF involves the application of electric field pulses usually at high voltages (kV range) and short durations (micro or nano-seconds) to a product placed between two electrodes [21]. The application of electric pulses produces the formation of reversible or irreversible pores in the cell membranes, defined as electroporation or electro-permeabilization, which consequently facilitates the rapid diffusion of the solvents and the mass transfer enhancement of intracellular compounds [22]. Recent applications have focused on the use of pulsed electric energy as an extraction technique (PEF-assisted extraction) from bio-, food, and agricultural products [23]. With PEF treatment it is feasible to obtain extracts with higher purity, increase the extraction rate of bioactive compounds such as polyphenols, carotenoids, or anthocyanins, and eliminate the use of organic solvents and to shorten the extraction time [24,25]. PEF treatment has been successfully applied for the extraction of valuable compounds from different marine sources, such as proteins [26,27,28], carbohydrates [29,30], lipids [31,32] and pigments such as carotenoids, chlorophylls or phycocyanins [22,33,34] from microalgae and seaweeds. 

Thus, the main objective of the present study was to assess the potential cosmetic applications of PEF extracts from three macroalgae species growing in Iceland: *U. lactuca* (green macroalgae), *A. esculenta* (brown macroalgae) and *P. palmata* (red macroalgae). In an effort, to develop organic and natural ingredients for green formulations, PEF-assisted extraction was proposed as an eco-friendly alternative to the traditional organic solvent extraction. After the extraction process, aqueous seaweed extracts were characterized in terms of polyphenol, flavonoid, and carbohydrate content. Moreover, antioxidant properties and enzymatic inhibitory activities were evaluated by using in vitro activity assays. Results reported herein will provide the basis for improving the understanding of brown, red, and green macroalgae to produce active ingredients for innovative formulations in cosmetic products containing biologically active compounds isolated from natural and sustainable sources.

## 2. Results and Discussion

### 2.1. PEF-Assisted Extraction for the Processing of Icelandic Seaweed Biomass

The results show that the electrical conductivity was highest in suspension prepared from *A. esculenta* followed by *P. palmata* and *U. lactuca* (*p* < 0.05) (Table 1). However, the effect of treatment type was not identified as significant (*p* > 0.05). Electrical conductivity measurement has been successfully used by other authors to evaluate the efficacy of PEF treatment in biological tissues for the release of intracellular ionic substances, as a result of the increased cell membrane permeabilization [35,36,37]. 

In our study, the results did not indicate a stronger release of these substances by PEF, since the changes in conductivity induced by extraction treatments tended to be highest in HW suspensions. Previous studies have concluded that the initial conductivity of the extracellular medium influences the electroporation efficacy but there is a lack of agreement of whether there positive or negative relationship between these two factors [38]. Variations in conductivity and characteristics of the material may make the comparison complicated. In our study, there was a large difference between conductivity of *A. esculenta* suspensions and the other two species, which was not reflected in the degree of conductivity changes during extraction treatment. It has been stated that ash content of brown seaweed can account for over 50 % of its dry weight [39], consisting largely of ions, which may partly explain the high conductivity in *A. esculenta* suspensions compared to the other two species.

The results show that the pH in *U. lactuca* suspension were lower than for the other two species, but no clear effects from extraction type were produced. The temperature was increased from 22 ± 1 °C before treatment, to 95 °C by HW (for all species), to 36.0 ± 1.0 °C, 46.3 ± 0.6 °C and 51.0 ± 1 °C by PEF, in *A. esculenta*, *P. palmata* and *U. lactuca* suspensions. The same trend was seen for the groups treated with PEF, which were then further heated by HW. The rise in temperature was caused by the conversion of electric energy to thermal energy (ohmic heating), in the suspension during PEF treatment. The level of temperature increase is known to be in proportion of to the applied current but in inverse proportion to the conductivity. This could explain why *P. palmata* and *U. lactuca* reached higher temperature during the PEF treatment although they have lower conductivity than *A. esculenta*. 

### 2.2. UV-VIS Absorption Spectra of Icelandic Seaweed Extracts

**Figure 1:**
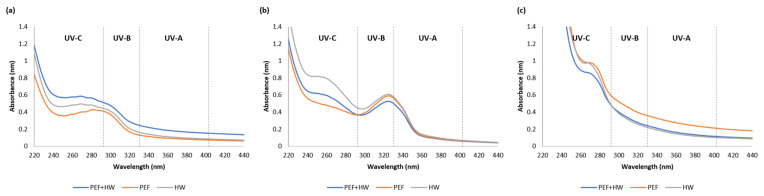
UV absorption spectra (λ = 220–440 nm) of seaweed extracts produced by hot water (HW), pulsed electric field (PEF) and the combination of both techniques (PEF + HW): *U. lactuca* (**a**), *P. palmata* (**b**) and *A. esculenta* (**c**). The graphs show the mean values (*n* = 3).

The studied seaweeds differ in the spectral profiles (Figure 1), suggesting that the composition and the UV-absorbance potential vary between species. However, the type of extraction technique did not exhibit a remarkable effect in the UV absorption spectra; seaweed extracts showed similar absorption profiles regardless of the extraction method.

The UV absorption spectra of the green alga *U. lactuca* showed a prominent peak in the UV-B range (280–320 nm) (Figure 1a), while the extracts from the brown alga *A. esculenta* showed no clear formation of absorption zone (Figure 1c). However, results indicated a stronger absorbance at 220 nm in *A. esculenta* extracts compared to *U. lactuca* and *P. palmata* which was presumed to results from the high content of phenolic compounds in *A. esculenta* (Table 2). An absorption maximum within this range has been related to a linkage between phenolic compounds and alginates. This relationship is presumed to preserve the UV absorption capability of phenolic compounds over time [40]. 

A more interesting finding was that the results obtained for the red algal extracts, *P. palmata* absorbed part of UV-A radiation (320–400 nm). It is known that red algae accumulate photoprotective compounds with ultraviolet radiation absorption capabilities such as mycosporine-like amino acids (MAAs), which absorb in this specific UV region [41]. *P. palmata* excelled in the UV absorption spectrum with prominent peaks between 320 and 340 nm in accordance with the presence of MAAs absorbing in this range [42], such as: palythinol (peak absorption at 332 nm), asterina-330 (absorption peak at 330 nm), porphyra-334 (peak absorption at 334 nm) and others [43]. Because extraction conditions, such as type of solvent, are known the influence the efficiency of extraction, the results in present study were compared to previous studies on the extraction of MAAs with water from *P. palmata.* In these studies, the absorption maximum peaks were detected at 325 to 330 nm [44], as in the present study. Therefore, it is possible to assume that the peaks observed between 320 and 340 nm may be due to the presence of MAAs. 

Differences in the absorption spectra between 350 and 700 nm have been explained by the presence of different accessory pigments in respective photosystems of green, brown, and red macroalgae, chlorophyll-b (450–500 nm), fucoxanthin (400–500 nm) and phycoerythrin (600–650 nm) respectively [45]. The concentration of water-soluble compounds in the extracts had stronger effects. Consequently, the pattern reflecting the difference in pigments between algae species was not apparent in the present study.

### 2.3. Total Phenolic, Flavonoid and Carbohydrate Content of Icelandic Seaweed Extracts

The total phenolic content in the seaweeds ranged from 1592 to 9368 µg GAE/g dw (Table 2). The brown alga *A. esculenta* showed the highest quantity (*p* < 0.05) of phenolic compounds (mean value 8869.7 µg GAE/g dw), followed by *P. palmata* (mean value 1806.2 µg GAE/g dw) and *U. lactuca* (mean value 1750.7 µg GAE/g dw) (there was no significant differences between *P. palmata* and *U. lactuca* extracts)). For each seaweed species, the content of polyphenols did not differ among extraction methods except for *U. lactuca*, which results showed that HW was the most efficient technique (*p* < 0.05). However, the advantages of PEF including its non-thermal nature, shorter extraction time (10 min vs. 45 min) and green process, should be highlighted.

Amongst the three algal groups, brown macroalgae contain a higher number of polyphenols than red and green macroalgae. Results were in agreement with early studies [46,47] who reported that brown (e.g., *A. esculenta* and *Saccharina latissma*) algae species had higher phenolic content than red (*P. palmata*) and green species (e.g., *U. lactuca*). This was supported by other authors [48] who concluded that the mean polyphenol content was species-specific (*A. esculenta* > *S. latissma* > *P. palmata*) and the phenolic content was more than three time higher in *A. esculenta* than in the other species (*A. esculenta*: 37 mg phloroglucinol equivalents (PGE)/g dw; *S. latissma*: 8 mg PGE/g dw; *P. palmata*: 5 mg GAE/g dw). Furthermore, in the same study, the authors reported that the polyphenol content varies with season, while the spatial variations (algae were harvested in Norway, France and Iceland) showed a marginal effect. For example, Gager et al. (2020) found that there was a significant effect of seasonal variations in polyphenol content of *A. esculenta*, with more than 300 mg GAE/g DW in autumn compared to under 20 mg GAE/g DW in springtime. Phlorotannins from seven brown seaweeds commercially harvested in Brittany (France) detected by 1 H NMR and in vitro assays: temporal variation and potential valorization in cosmetic applications. Our samples were collected in July (*U. lactuca* and *A. esculenta*) and in November (*P. palmata*). In Roleda’s study [48], the average content in *A. esculenta* from Trondheim, Norway (not collected in Iceland) in summer was 40 mg PGE/g dw and *P. palmata* from Iceland but was 4 mg GAE/g dw in the autumn. The higher values reported in comparison with our study can be explained by the extraction media used (80:20 acetone:water), likely to result in higher extraction yields. A higher polyphenol content was also found for *A. esculenta* extracts using a mixture of ethanol and water (50:50) with ultrasound [49]. However, using the same extraction medium and the classic solvent extraction, *A. esculenta* was reported to contain 44.1 mg GAE/100 g dw in aqueous extracts [50], relatively similar to that observed in the present study.

Mean flavonoid content was species-specific (*A. esculenta* > *U. lactuca* > *P. palmata*; (*p* *<* 0.05) (Table 2). The highest amount of flavonoids was observed for *A. esculenta* extracts (mean value 12098.7 µg QE/g dw), while lower content was found for *U. lactuca* (mean value 4152.4 µg QE/g dw), and a minimum content was determined for *P. palmata* extracts (mean value 905.8 µg QE/g dw). Similar to the behaviour found for the total phenolic content, the type of extraction technology did not have significant effects on the flavonoid content (*p* > 0.05), with the exception of *U. lactuca*. Results showed that HW and the combination of both techniques (PEF + HW) were the most efficient techniques for the extraction of flavonoids in *U. lactuca* (*p* < 0.05). 

There are numerous studies on the flavonoid content in terrestrial plants, but flavonoid content studies in algae are scarce [51] and especially in the species studied in the present work. Namely, the study of Ummat et al. [49] reported that ultrasound assisted extraction enhanced the recovery of flavonoids in all 11 seaweeds investigated (including *A. esculenta*) compared to conventional solvent extractions using a mixture of 50% ethanol. In another study, flavonoids were quantified in the methanolic extracts of four *Ulva* species (*Ulva clathrata*, *Ulva linza*, *Ulva flexuosa* and *Ulva intestinalis*) grown at different parts of northern coasts of the Persian Gulf in south of Iran; the flavonoid content of algal extracts varied from 8 to 33 mg RE/g dw [52]. However, previous studies by the same research group found marked changes in the chemical constituents with change of seasons and environmental conditions [53]. Thus, it is a little hard to have a full overview of the bibliography of these bioactive compounds in seaweeds, due to the lack of available published research, but also because of the changes in the flavonoid content influenced by the growing conditions and geographic location.

Mean carbohydrate content of produced extracts was also species-specific (*P. palmata* > *U. lactuca* > *A. esculenta*; *p* < 0.05) (Table 2). Contents ranged from 44.8 to 510 mg GluE/g dw depending on algae species. Seaweed contains large amount of polysaccharides with important functions for the macroalgal cells including structural support and energy storage. For instance, the main part of red and brown seaweed cell walls is represented by sulfated galactans, which are known as agar, alginate, and carrageenan [54]. The red algae *P. palmata* showed the highest amount of carbohydrate content (mean value 441 mg GluE/g dw). Results were in agreement with previous studies that reported the highest polysaccharide concentration in *Palmaria* species [55]. Moreover, Mutripah et al. [56] described a total carbohydrate content of *P. palmata* of 469 mg/g of dry seaweed, relatively similar to that observed in the present study. 

The green macroalgae *U. lactuca* showed contents of up to 249.5 mg GluE/g dw depending on the extraction technique used (Table 2). Based on literature, *U. lactuca* has water-soluble and insoluble cellulose corresponding to structural polysaccharides with a major component called ulvan, which contributes from 9 to 36% dry weight the biomass [57]. Ulvan is mainly composed of sulfated rhamnose, uronic acids (glucuronic acid and iduronic acid) and xylose. Due to its polar nature, the solubility of ulvan in aqueous solutions is enhanced by extraction at high temperatures (80–90 °C) [58]. The extraction temperature could be the reason why the total carbohydrate content of *U. lactuca* extracts produced by the traditional hot water extraction and the combination of both methods (PEF + HW) was higher (*p* < 0.05) than the content achieved using only PEF. 

On the other hand, other authors highlight the importance of the seasonal variation in the polysaccharide content. For instance, Schiener et al., claim to identify seasonal variations and predict best harvest times for kelp. The seasonal composition analysis of *A. esculenta* demonstrated that maximum values of carbohydrates coincided with reduced concentrations of protein, ash, polyphenols and moisture [39]. According to the authors, these relationships, which vary between seasons and species, can be used by industries to maximize yields of targeted seaweed components.

### 2.4. Antioxidant Capacities of Icelandic Seaweeds Extracts

*A. esculenta* had the strongest DPPH scavenging activity among the crude extracts of the three algae species (*p* < 0.05), with scavenging effect higher than 90% (Table 3). Compared with the different standard solutions, *A. esculenta* showed comparable scavenging activity as 100 µg/mL of ascorbic acid (87.9%), gallic acid (91.0%) and α-tocopherol (87.9%). Our results were in agreement with recent studies [50], which also reported a positive antioxidant activity of *A. esculenta* extracts. Surprisingly, no significant differences in antioxidant activity were observed between the different extraction methods tested (*p* > 0.05). It was expected that PEF extracts would show better antioxidant values than the extracts produced with the hot traditional extraction since other studies have shown that green techniques (such as microwave assisted extraction or enzymatic extraction) could effectively avoid the decomposition of bioactive compounds, exhibiting higher antioxidant activities [59,60]. 

The ability of seaweed extracts to reduce ferric (Fe^3+)^ to ferrous (Fe^2+^) ion and the ability to scavenging the radical ABTS was also studied, by the FRAP and ABTS method, respectively. FRAP results showed similar trends to DPPH, showing *A. esculenta* had the strongest ability to reduce ferric (Fe^3+^) to ferrous (Fe^2+^) ion among the crude extracts of the three algae species (*p* < 0.05). However, a different behavior was found for the ABTS. All seaweeds extracts showed similar ability to scavenging the radical ABTS (*p* > 0.05), indicating that these species probably contain some efficient compounds which are responsible for its scavenging activity. 

In general, brown algae are known to present higher antioxidant potential in comparison to red and green families [61]. Our results also showed that aqueous extracts from *A. esculenta* exhibited effective antioxidant activities with regards to the scavenging of free radicals and reducing power, suggesting that *A. esculenta* could potentially be a resource for natural antioxidants. The high antioxidant activity observed for *A. esculenta* extracts could be linked to the high content in phenolic compounds determined in the brown algae extracts. In many studies, the antioxidant activity of algae extracts has been ascribed to the phenolic compounds, showing positive correlations between phenolic content and scavenging capacity mostly with DPPH [62,63]. Similar correlation results were found in the current study for *A. esculenta* extracts (see a better discussion in Section 2.6. Correlations between chemical compounds and bioactive properties). 

### 2.5. Enzymatic Inhibitory Activities of Icelandic Seaweed Extracts

Icelandic seaweeds extracts exhibited positive inhibitory effects towards all enzymes tested (Table 4), opening new avenues for the exploitation of natural enzymatic inhibitors from algae resources. To the best of our knowledge, this is the first time that enzymatic inhibitory activities of Icelandic seaweed extracts produced by PEF have been tested.

#### 2.5.1. Collagenase Inhibition Activity

*A. esculenta* extracts showed positive collagenase inhibition ranging from 68 to 91%, while *P. palmaria* and *U. lactuca* extracts exhibited insignificant inhibition activities against collagenase (Table 4). *A. esculenta* hot water extract exhibited 71.1% collagenase inhibition activity, which was higher than epigallocatechin-3-gallate (EGCG) standard solution (63.2%) and comparable with positive standard providing by the commercial enzymatic kit (74.9%). An important finding was that the *A. esculenta* extracts produced by the PEF showed a collagenase inhibition of 91%, exhibiting even higher activity than the inhibitor provided by the commercial kit. It should be highlighted that this activity was only observed in the water extracts produced by PEF and not by the combination of PEF+HW. This behavior can be explained by the possibility that the hot water process could have a negative effect on the compounds responsible for inhibiting the collagenase activity. However, additional studies are needed to explain these results due to the complexity of crude algal extracts. The aforementioned research group is currently working on the identification of the inhibition molecules in *A. esculenta* extracts to better understand these positive effects produced by the PEF. 

Results regarding the inhibition of collagenase by *A. esculenta* extracts are in accordance with previous data, in which *A. esculenta* is being used in commercial extracts due to its antiaging effect. The degradation of collagen occurs with aging due to collagenase activity, resulting in wrinkles on the skin. The inhibition of collagenase by naturally occurring compounds is an interesting opportunity for anti-aging products. For instance, SEPPIC, a supplier of ingredients for the cosmetics industry, is offering a lipophilic extract of *A. esculenta* (Kalpariane^®^ AD) [64]. 

#### 2.5.2. Elastase Inhibition Activity

Only the crude extracts of *A. esculenta* inhibited elastase, exhibiting activities higher than 70% of inhibition (Table 4). However, the anti-elastase activities of *A. esculenta* extracts did not statistically differ among extraction methods (*p* > 0.05). Compared with quercetin solutions, a well-known elastase inhibitor that showed 100% inhibition at 1 mM and 58.7% at 0.5 mM, the performance of extracts from *A. esculenta* was high. 

Elastase is a proteinase enzyme that can reduce elastin by breaking specific peptide bonds. Consequently, the inhibition of elastase activity in the dermis layer can be used to maintain skin elasticity [65]. Many plant extracts have been identified as elastase inhibitors [17]; however, few investigations have been carried out on the elastase inhibition from algae resources. According to literature data, polyphenols extracted from plants are known to be strong elastase and hyaluronidase inhibitors [66]. A recent study reported that the phlorotannins, the type of tannin in brown algae, extracts of sea kelp *Eisenia bicyclis* and brown alga *Ecklonia cava,* benefit the skin by reducing the elastase activity significantly [67]. The *A. esculenta* extracts produced in this study showed the highest TPC and TFC values in comparison to the other species studied (Table 4), so this could be the reason why the aqueous extracts from *P. palmaria* and *U. lactuca* did not show anti-elastase activities. To confirm this hypothesis, Pearson correlation analysis was conducted, suggesting that the anti-enzymatic activities positively correlate with the content of phenolic substances (see a further discussion in Section 2.6. Correlations between chemical compounds and bioactive properties).

#### 2.5.3. Tyrosinase Inhibition Activity

*A. esculenta* extracts showed positive tyrosinase inhibition higher than 90% for all the extraction methods used, while *P. palmaria* and *U. lactuca* extracts did not exhibit tyrosinase inhibitory effects (Table 4). However, the anti-tyrosinase activities of *A. esculenta* extracts did not differ (*p* < 0.05) with extraction methods. Comparing the effect of *A. esculenta* extracts with the quercetin solutions tested, the crude extracts of the brown algae showed better inhibitory activities than these solutions (88 and 75% for the 0.5 and 1 mM quercetin solutions, respectively). Based on literature, anti-tyrosinase activities of plants, bacteria and fungi have been reported by several researchers [68]. However, though different studies suggest that bioactive compounds derived from marine algae have a good potential to be utilized as skin whitening agents [13], this is still an unexplored domain and only a few studies have been carried out. Most of the studies performed in this area have been focused on brown algae, agreeing with the results of the present study in which *A. esculenta* extracts exhibited the best anti-tyrosinase activities. For instance, phloroglucinol derivatives and phlorotannins, common secondary metabolites found in brown algae, have shown inhibitory activity against tyrosinase due to their ability to chelate copper [69]. In a recent study, the extract of the brown algae *Lessonia trabeculate produced by microwave-assisted extraction* inhibited a tyrosinase activity of 33.73% [60]. In another study, the extract of the brown algae *Turbinaria conoides* showed activity as an antioxidant and tyrosinase inhibitor, however, in this case ethanol was used as solvent [70]. A significant correlation between the inhibitory potency of polyphenols extracted from plants on mushroom tyrosinase has been reported in previous studies [68]. Likewise, the results of this study suggest that the inhibitory activity towards tyrosinase were positively correlated with flavonoid and phenolic content (see Section 2.6. Correlations between chemical compounds and bioactive properties).

Tyrosinase plays an important role in the biosynthesis of melanin pigment in skin. Melanin is responsible for the protection against harmful ultraviolet irradiation, which can cause several pathological conditions [71]. In addition, it can create aesthetic problems when melanin is accumulated as hyperpigmented spots [72]. Thus, incorporating tyrosinase inhibitors in cosmetic products can be attractive due to whitening and or lightening effects. 

#### 2.5.4. Hyaluronidase Inhibition Activity

All the seaweeds extracts exhibited significantly high anti-hyaluronidase activity (Table 4), showing comparable results to the tannic acid solutions (a well-known inhibitor of hyaluronidase). Specifically, *A. esculenta* extracts showed 100% of inhibition for all the methods tested. Moreover, *U. lactuca* extracts exhibited activities higher than 90% of inhibition, where the inhibition of the extracts produced by PEF (96.8%) and the combination of PEF + HW (97.3%) was higher than the inhibition produced by the traditional hot water method 93.4%) (*p* < 0.05). All *P. palmaria* extracts exhibited similar activities (*p* < 0.05), the inhibition of the extracts produced by PEF was (91.9 %) and the combination of PEF + HW (89.5%) and the traditional hot water method (91.8%).

Other authors also described anti-hyaluronidase activity of different seaweeds extracts, especially for extracts rich in phlorotannins from brown algae [73,74]. However, to the best of our knowledge, this is the first time that hyaluronidase inhibitory activities of *P. palmata* and *U. lactuca* extracts produced by PEF have been reported. 

Hyaluronic acid is a major component of the dermis, where it is involved in tissue repair, it breaks down with aging, causing wrinkles and loss of skin firmness. In this sense, hyaluronidase inhibitors increase the hyaluronic acid level of the dermal extracellular matrix for the improvement of the appearance of aging facial skin [13]. Therefore, the results of this study might open new avenues for the exploitation of natural hyaluronidase inhibitors from algae resources with potential use in cosmetic products.

In summary, the data gathered allowed us to conclude that *A. esculenta* extracts exhibited in overall better inhibitory activities than *P. palmaria* and *U. lactuca* towards the enzymes tested. Thus, being the most promising seaweed specie with excellent anti-enzymatic activities and therefore it was selected for further studies in our laboratory. Although crude extracts from *A. esculenta* appear to be good candidates in in vitro experiments, further studies need to be carried out to elucidate the identity of the metabolites responsible for these biological effects. 

### 2.6. Correlations between Chemical Compounds and Bioactive Properties

**Figure 2:**
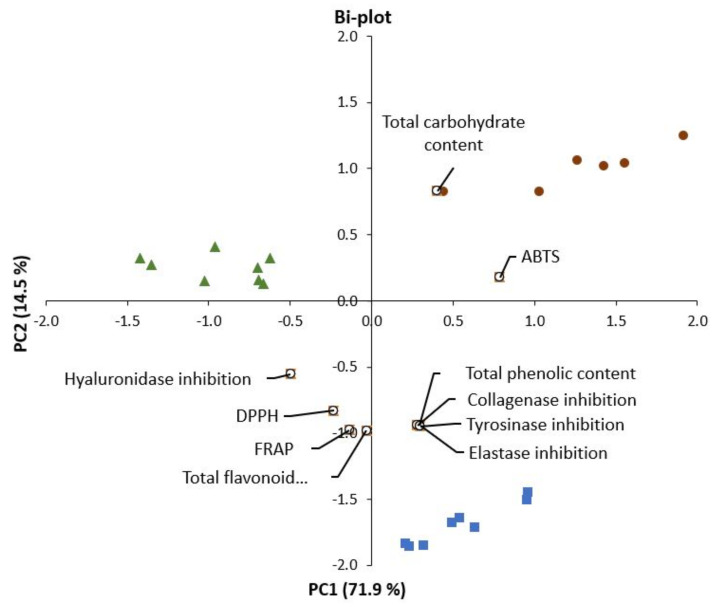
Principal component analysis (PCA) biplot of factor loadings (black circles) and scores for the algae extracts (*P. palmata*: brown dots (upper right); *U. lactuca*: green triangles (mainly in upper left); *A. esculenta*: blue squares (lower part of plot)).

The results from principal component analysis (PCA), showed that the main separation of the groups was defined by PC1 and PC2, which accounted for 71.9% of and 14.5% of variance in the data, respectively (Figure 2). The *A. esculenta* extracts were characterized by higher contents of flavonoids and phenolic compounds, inhibitory effects on enzymes (collagenase, tyrosinase and elastase), and DPPH and FRAP values, than to the other species, *P. palmata* and *U. lactuca*. On the other hand, *A. esculenta* had lower carbohydrate content, especially compared to *P. palmata* (which was located at the opposite side of the PC1). The variation in data along the PC2 was mainly related to ABTS and hyaluronidase inhibition. As indicated by the location on the plot, *P. palmata* had a stronger correlation to ABTS whereas *U. lactuca* was more related to hyaluronidase inhibition effects, in comparison of these two species.

High and significant positive correlation between TPC, TFC, DPPH, FRAP, and inhibitory effects on collagenase, elastase and tyrosinase was demonstrated by Pearson correlation analysis (Table 5). 

This was in an agreement with previous studies, reporting that phenolic compounds (including flavonoids) are the main contributors to antioxidant activity of various seaweeds [75,76,77]. The high antioxidant activity of extracts from brown macroalgae has been related to a specific group of polyphenols, phlorotannins, and their unique molecular structure. Phlorotannis from brown algae are reported to have up to eight interconnected phenol rings that act as electron traps [78,79]. It was expected that ABTs would correlate with TPC, other antioxidant parameters. Possible reasons might be that the methods are based on different reaction conditions and that reactivity differs both with regards to time and range of components. For example, the ABTS reagent reacts with broader range of antioxidants than the DPPH radical [80]. On the other hand, one of the limitations mentioned for ABTS is a long reaction and the general reaction time may not allow reaching an endpoint. 

The results indicate that there is a high positive correlation of TPC and TFC to the inhibitory activity of collagenase, elastase and tyrosinase (0.93–0.99), whereas the relationship to inhibition of hyaluronidase was not as strong (r = 0.42 and 0.54, respectively). This indicates that other components may have contributed to the inhibitory effect of the extracts. Other studies have reported the polysaccharides have hyaluronidase-inhibitory activity, for instances alginic acid in brown algae [81,82]. Further studies on the chemical composition of the macroalgae species for on the effects of isolated compounds on the enzyme is needed to evaluate the contribution of each chemical component as in this study the focus was on crude extracts. 

The findings were in harmony with previous studies, stating that the chemical composition and levels of bioactivity of the extracts vary significantly between the three linages (red, green and brown algae) and between different species belonging to the same phylum and are influenced by age and tissue type. Furthermore, the composition and characteristics depend on many environmental factors affecting the distribution and growth of macroalgae. For example, light (UV-radiation), temperature, nutrient availability, exposure to air, water motion, wave exposure and salinity. Temperature has been described as the factor having the strongest effects on pigment formation and nutrient concentration, salinity, and UV radiation as the factors influencing the concentration of TPC [83].

The distribution of different macroalgae species varies with water depth. Positions higher up the shore in the intertidal or littoral zone are more stressful as the species growing there, must withstand multiple changes in abiotic factors due to tidal changes. For instance, the drying effect of air, high solar irradiances (at low tide), changes in salinity and temperature and, under conditions of low air temperatures, including freezing. Below the low water mark, increasing depth results in a very rapid decrease in light intensity and less exposure to irradiance. 

Algae growing in the tidal range have lower sensitivity to UV Radiation and recovery more rapidly form solar stress. Whereas algae growing in the sublittoral zone are more sensitive to UV radiation and have lower recovery from solar stress [84]. At the same time the water column provides protection. In the present study the exposure to sunlight was presumably stronger for *P. palmata*, compared to the other species. Other studies have shown that formation of MAAs is directly related to sunlight [85], protecting the organisms against UV-A and UV-B radiation. Moreover, it was shown that the specific amount of MAAs decreased with increasing collecting depth. Kelps such as *A. esculenta*, are known to grow at the upper sublittoral zone but also extend in to the lowest intertidal just above the low water mark. Meaning water column provided stronger protection than for *P. palmata*. In addition, the morphological characteristics are different, the blades of *A. esculenta* are thicker compared to the other two species. *U. lactuca*, growing mainly in the intertidal and sublittoral is able to photosynthesize and grow under very low irradiances. Exposure to UVB light has been stated to accelerate the recovery of photosynthetic parameters of *U. lactuca* from the negative effects of UVA light. It is smaller, simpler in structure and shorter lived (3 months) than both *A. esculenta* (5–7 year) and *P. palmata* which has a new growth every year. 

In summary, the assumptions can be drawn that the main differences in the properties of the extracts are to the variation in life span, morphological characteristics and growth conditions of the algae species. 

## 3. Materials and Methods

### 3.1. Materials

Icelandic seaweeds *U. lactuca* (green algae), *A. esculenta* (brown algae) and *P. palmata* (red algae) were provided by Icelandic Blue Mussel and Seaweed, which harvested seaweeds in Breidafjordur (West-Iceland). After harvesting the seaweeds were dried (to approximately 90% dry material), milled and delivered vacuum packed. Samples were kept in a dry and dark place at room temperature until used.

Tyrosinase from mushroom, L-3,4-dihydroxyphenylalanine (L-DOPA), elastase from porcine pancreas, ascorbic acid, N-Succinyl-Ala-Ala-Ala-*p*-nitroanilide (AAAPVN), hyaluronidase from bovine testes, quercetin, α-tocopherol, tannic acid, 2,2-diphenyl-1-picrylhydrazyl (DPPH), 2,4,6-Tripyridyl-s-Triazine (TPTZ), Trolox, Folin-Ciocalteu reagent, gallic acid and a collagenase activity colorimetric assay kit (MAK293) were purchased from Sigma-Aldrich Co. (St. Louis, MO, USA). Hyaluronic acid sodium salt was purchased from MakingCosmetics (Redmond, WA, USA). All other chemicals and reagents used were analytical grade and obtained from VWR International, LLC. Deionized water (Elix^®^ Essential, Merck, Darmstadt, Germany) was used for the extraction and preparation of water-based solutions.

### 3.2. Experimental Design

Factorial design was used for evaluating effects of Icelandic seaweed species (*U. lactuca*, *A. esculenta*, *P. palmata*) and extraction treatment (hot water extraction (HW, 95 °C)), PEF-assisted extraction (PEF) and the combination of both techniques (PEF + HW), on extract composition and bioactivity (Table 6). The extraction was carried out in triplicate for each group and every extract replicate was analyzed in triplicate.

### 3.3. The Extraction of Bioactives from the Icelandic Seaweeds

The exploitation of macroalgal biomass at different levels has motivated scientists to explore more eco-friendly, efficient, and cost-effective extraction techniques, based on green extraction approaches. In this work, PEF-assisted extraction was evaluated as a novel and green method to produce functional extracts, while traditional hot water extraction was used for comparison. Moreover, the effect of the combination of both techniques, PEF treatment of macroalgae followed by the traditional hot water extraction, on the bioactive recovery was studied. Due to the expected electroporation produced in the cell membranes after the physical treatment, the following extraction with hot water could further facilitate the release of the intracellular material [86], increasing the extraction yield. A time after treatment is needed for the materials to diffuse out of the cells [87,88], and in this experiment the suspensions waited overnight until separation of the liquid (extract) from the pulp. 

Regarding extraction medium, distilled water was used to produce the seaweed extracts to overcome limitations concerning the use of toxics and organics solvents. Water proved to be a good solvent for the extraction of several bioactive compounds from seaweeds [46,89,90,91], and is environmentally friendly. In addition, water is commonly used for PEF-assisted extraction as it is a good conductor for electricity.

#### 3.3.1. Extraction Procedures

For every replicate in each group, seaweeds (15 g) were soaked overnight at room temperature (22 °C) in deionized water (300 mL). Then, the suspension was treated with PEF (PEF), heated (HW) or both PEF-treated and heated (PEF+HW). The suspensions were kept over-night in a refrigerator followed by filtration with a coarse (20 µm) filter paper. Then the filtrates (extracts) were stored at 4 °C until their analyses. 

The pulsed electric fields-assisted extraction was carried out by using a pulse generator built inhouse. It had a F.u.G.HCK-200-2000 capacitor (F.u.G. Elektronik GmbH, Rosenheim, Germany) and spark gap (18.5 kV OG75, Perkin-Elmer Optoelectronics, GMBH, Wiesebaden, Germany). The PEF equipment generated exponential decay pulses with a width of 0.96 µs and amplitude of 18 kV. A plexiglass treatment chamber with the dimensions (L × H × W) 20 × 8 × 2.5 cm, with the shortest distance being between the plate electrodes was used treating the suspensions with an 8 kV/cm electric field at 1.2 Hz for 10 min.

The HW-extracts were prepared by heating the suspension in a beaker in a thermostatic water bath and kept at 95 °C for 45 min. For the combined pulsed electric field and heating treatment, the suspensions were PEF treated and then placed in a beaker, heated in a water bath, and kept at 95 °C for 45 min.

#### 3.3.2. Conductivity, pH and Temperature Measurements

The electrical conductivity and pH of seaweeds suspensions were measured after soaking and after the extraction treatments, at room temperature, using a pH-meter (Orion Star™ A215 pH/Conductivity Benchtop Meter, Thermo Scientific, Waltham, MA, USA) equipped with a conductivity sensor and pH/ARC triode combination electrode. Furthermore, temperature changes due to treatments were recorded.

### 3.4. Spectral Profiles of the Seaweed Extracts

The UV-VIS absorption spectra of the different seaweed extracts were measured for the range of 200 to 450 nm using a double beam Thermo Scientific Evolution 350 UV-Vis Spectrophotometer (Thermo Fisher Scientific, Waltham, MA, USA) with 1 cm quartz cuvettes. Three scans were performed for each seaweed extract.

### 3.5. Determination of Total Polyphenolic Content

The total phenolic content (TPC) in seaweed extracts was determined by using Folin–Ciocalteu reagent following a slightly modified method described by Zhang [92] using a Multiskan Sky Microplate Spectrophotometer (Thermo Fisher Scientific, Waltham, MA, USA). A volume of 20 µL of seaweed extract or serial standard solution was mixed with 100 µL of Folin–Ciocalteu reagent (10% in distilled water). After 5 min, 80 µL of 7.5% (*v*/*w*) sodium carbonate solution was added. The reaction mixture was incubated at room temperature and darkness for 30 min. Absorbance was measured at the wavelength of 760 nm. Distilled water was used as blank. A standard curve of gallic acid was used to determine the total phenolic content and expressed as µg of gallic acid equivalents (GAE) per gram of dry material (µg GAE/g dw). 

### 3.6. Determination of Total Flavonoid Content

The total flavonoid content (TFC) in seaweed extracts was determined by the method described by Kamtekar [93] and adapted to 96-well microplates. Briefly, a volume of 25 µL of seaweed extract or serial standard solution was mixed with 100 µL of sodium nitrite (0.375% *w*/*v*). After 5 min, 25 µL of aluminum chloride (3% *w*/*v*) was added to the mixture and incubated for 6 min at room temperature. Then, 100 µL of sodium hydroxide (2% *w*/*v*) was added to the mixture and mixed. Immediately, absorbance was measured at the wavelength of 510 nm. Distilled water and ethanol were used as blanks. A standard curve of quercetin (dissolved in ethanol) was used to determine the total phenolic content and expressed as µg of quercetin equivalents (QE) per gram of dry material (µg QE/g dw). 

### 3.7. Determination of Carbohydrate Content

The free sugars content was measured according to the method described by [94], with slight modifications. A 50 µL of phenol solution (4%) and 250 µL of sulfuric acid (96%) were added to 100 µL of sample or standard solution. After 10 min of incubation at room temperature, the absorbance of the mixture was read at 490 nm. A standard curve of glucose was used to determine the total carbohydrate content and expressed as mg of glucose equivalents (GluE) per gram of dry material (mg GluE/g dw). 

### 3.8. Antioxidant Properties of Seaweeds Extracts

#### 3.8.1. 2,2 Diphenyl-1-picrylhydrazyl (DPPH) Free Radical Scavenging Assay

The antioxidant activity (DPPH) of seaweed extracts was determined following the previously described methodology [94] with some modifications. Briefly, 200 μL of 10.825 × 10^−5^ M DPPH solution was added to 100 μL of sample (1:1 in methanol) in a 96-well plate. The same volume of DPPH was mixed with 50 μL standard +50 μL methanol. Then the samples and standard were incubated in a dark place at room temperature for 30 min. The absorbance was measured at the wavelength of 517 nm. Distilled water was used as a blank. The ability to scavenge the DPPH radical was calculated using the follow equation:

Scavenging effect (%) = (1 − (A_sample_ − A_sample blank_)/(A _control_ − A_methanol blank_)) × 100 (1)

where A_control_ is the absorbance of the control (DPPH solution without sample), the A_sample_ is the absorbance of the test sample (DPPH solution plus test sample), the A_sample blank_ is the absorbance of the sample only (sample without DPPH solution) and A_methanol blank_ is the absorbance of methanol only. Commercial antioxidants (ascorbic acid, gallic acid and α-tocopherol) were used as positive controls. 

#### 3.8.2. Ferric Ion Reducing Antioxidant Power (FRAP) Assay

FRAP activity was measured according to the method of Benzie and Strain [95]. Briefly, acetate buffer (300 mM, pH 3.6), 2,4,6-tripyridyl-s-triazine (TPTZ) 10 mM in 40 mM HCl, and FeCl_3_·6H_2_O (20 mM) were mixed in the ratio of 10:1:1 to obtain the working FRAP reagent. The reaction mixture was incubated at 37 °C for 10 min. A 50 μL sample from every extract was mixed with 150 μL of working FRAP solution for 8 min at room temperature. The absorbance of colored product, Ferrous-TPTZ was measured at the wavelength of 593 nm. FRAP values of seaweeds extracts were expressed as µM of trolox equivalents (TE) per gram of dry material.

#### 3.8.3. 2,2 Azino-bis(3-ethylbenzothiazoline-6-sulfonic Acid) (ABTS) Assay

The analysis was performed using the ABTS decolorization protocol [76] with some modifications. A ABTS radical cation (ABTS^.+^) was produced by reacting ABTS (66 mg) with 10 mL of potassium persulphate solution (2.45 mM). The mixture was left in the dark at room temperature for 12–16 h before use. The ABTS^.+^ solution was diluted with water to an absorbance of 0.700 at 734 nm. The reaction mixture (200 ul) was transferred to a microplate, 50 µL of sample was added and then 150 µL of the reagent solution. The plate was shaken for 10 s at medium speed, and the absorbance was measured at 734 nm after 5 min of incubation at room temperature. A standard curve was prepared by plotting the inhibition of A_734nm_ of Trolox standards as function of their concentrations. The Trolox equivalent antioxidant capacity (TEAC) value of the samples was calculated using the equation obtained from the linear regression of the standard curve substituted of A_734nm_ values for each sample:

TEAC (µM) = (sample inhibition A_734nm_ − intercept)/slope (2)

The antioxidant activity was expressed in terms of TEAC concentration, µmol/g dry weight algae.

### 3.9. Anti-Enzymatic Activities of Seaweeds Extracts

#### 3.9.1. Collagenase Inhibition Assay

A collagenase activity colorimetric assay kit (MAK293), purchased from Sigma-Aldrich, was used to determine the collagenase inhibition of seaweeds extracts. The kit measured collagenase activity using a synthetic peptide (FALGPA) that mimics collagen’s structure. The procedure was performed according to the kit instructions. 

#### 3.9.2. Elastase Inhibition Assay

The elastase inhibition of seaweeds extracts was investigated in TRIS buffer solution with modified method as described earlier [96]. Briefly, 100 µL of 0.1 M TRIS buffer solution (pH 8.0), 25 µL of elastase (1 U/mL in TRIS buffer) and 25 µL sample extracts were mixed and incubated for 15 min at 30 C before adding the substrate to begin the reaction. After incubation time, 50 µL of 2 mM AAAPVN solution was added. Then, the absorbance at 420 nm was monitored for 20 min using a microplate reader under constant temperature of 30 C. Finally, elastase inhibition was calculated in percentage using the equation:

% Inhibition = [(ΔAbs/min_control_ − ΔAbs/min_sample_)/ΔAbs/min_control_] × 100 (3)

where, Abs_control_ is the absorbance of the assay using the buffer instead of inhibitor (sample) and Abs_sample_ is the absorbance of the sample extracts. Quercetin was used as positive control. TRIS buffer was used as blank. 

#### 3.9.3. Tyrosinase Inhibition Assay 

Tyrosinase inhibitory assay was performed according to the method previously described by [66] using L-DOPA as substrate. A 20 µL of sample, 10 µL of mushroom tyrosinase solution (50 U/mL in phosphate buffer) and 80 µL of phosphate buffer (pH = 6.8) were mixed in a microplate and pre-incubated at 37 °C for 5 min. Then, 90 µL of L-DOPA (2 mg/mL) was added. The formation of dopachrome was immediately monitored for 20 min at 475 nm in a microplate reader under constant temperature of 37 °C. The percent inhibition of tyrosinase enzyme was calculated using the equation:

% Inhibition = [(ΔAbs/min_control_ − ΔAbs/min_sample_)/ΔAbs/min_control_] × 100 (4)

where, Abs_control_ is the absorbance of the assay using the buffer instead of inhibitor (sample) and Abs_sample_ is the absorbance of the sample extracts. Quercetin was used as positive control. Phosphate buffer was used as blank. 

#### 3.9.4. Hyaluronidase Inhibition Assay

Hyaluronidase inhibitory activity was measured as previously described by [66] with few modifications. A volume of 100 μl of type-1-S bovine testes hyaluronidase (2100 U/mL) dissolved in 0.1 M acetate buffer (pH 3.5) was mixed with 100 μL of extract, and incubated at 37 °C for 20 min. A volume of 200 μL of 6 mM of calcium chloride was added to the reaction mixture, and then the mixture was incubated at 37 °C for 20 min. This Ca2+ activated hyaluronidase was treated with 250 μL of sodium hyaluronate (1.2 mg/mL) dissolved in 0.1 M acetate buffer (pH 3.5), and then incubated in a water bath at 37 °C for 40 min. A 50 μL of 0.9 M sodium hydroxide and 100 μL of 0.2 M sodium borate were added to the reaction mixture, and then incubated in a boiling water bath for 5 min. After cooling to room temperature, 250 μL of ρ-dimethylaminobenzaldehyde (DAMB) solution was added to the reaction mixture. The DAMB solution was prepared by dissolving 0.25 g of DAMB in 21.88 mL of 100% acetic acid and 3.12 mL of 10N hydrochloric acid. The control group was treated with 100 μL of 5% of water instead of extract. The absorbance was measured at the wavelength of 585 nm after 45 min. The percentage enzyme inhibition was calculated using the following equation:

% inhibition = [(Abs_control_ − Abs_sample_)/Abs_control_] × 100 (5)

where, Abs_control_ is the absorbance of the assay using the buffer instead of inhibitor (sample) and Abs_sample_ is the absorbance of the sample extracts. Tannic acid is used as a reference standard.

### 3.10. Statistical Analysis

The average of the triplicate analysis of every extract was calculated and used to find the mean values and standard deviations for each group (*n* = 3). General linear models (GLM) for fixed factors were applied to evaluate main effects and two-way interactions of the experimental factors (species and extraction methods) on measured variables. Furthermore, ANOVA and the Tukey–Kramer test was used to identify significant (*p* < 0.05) differences between the groups. Pearson correlation was used to evaluate linear relationship between the variables. Principal component analysis (PCA) was used to detect structure in the relationship between measured variables and experimental factors. The PCA reduces voluminous data to a small set of linear combinations of related variables (i.e., factors) based on patterns of correlation among the original variables. The resulting linear attribute combinations can be used for profiling specific product characteristics based on the variables studied. All statistical analyses were performed using NCSS 2020 Statistical Software (2020) (NCSS, LLC., Kaysville, UT, USA).

## 4. Conclusions

The outcomes of this first screening experiment showed the potential of three Icelandic seaweed species by providing effective beneficial effects via several pathways. The green approach developed using aqueous pulsed electric fields exhibited similar results to the traditional hot water extraction, showing several advantages such as its non-thermal nature and shorter extraction time (10 min vs. 45 min). Amongst the three algal species, the brown macroalgae *A. esculenta* showed the highest content of TPC and TFC also exhibiting the greatest antioxidant capacities Moreover, *A. esculenta* water extracts exhibited better inhibitory activities than *P. palmaria* and *U. lactuca* towards collagenase, elastase, tyrosinase and hyaluronidase being the most promising seaweed specie with excellent anti-enzymatic activities for their use in skin whitening, anti-aging and skin health. Interestingly, the *A. esculenta* extracts produced by PEF method showed a collagenase inhibition of 91%, higher than the inhibition activity showed by the traditional hot water extraction and even higher than the inhibitor provided by the commercial kit. In conclusion, our preliminary study suggests that Icelandic seaweed-based extracts, especially the extracts from the brown macroalgae *A. esculenta*, produced by aqueous pulsed electric fields-assisted extraction are potential functional ingredients that could be used as active compounds for cosmetic and cosmeceutical formulations in the near future.

## Figures and Tables

**Table 1:** Electrical conductivity and pH of seaweed suspensions before and after extraction process, and temperature after process.

Seaweed Specie and Extraction Method	T (°C)	Conductivity before (mS/cm)	Conductivity after (mS/cm)	pH before	pH after
***A. esculenta***					
HW	95.0	16.433 ± 0.260 ^a^	18.413 ± 0.228 ^a^	6.49 ± 0.02 ^a^	6.28 ± 0.05 ^a^
PEF	36.0	16.790 ± 0.131 ^a^	17.713 ± 0.091 ^a^	6.37 ± 0.01 ^b^	6.32 ± 0.01 ^a^
PEF+HW	34.3/95	16.560 ± 0.259 ^a^	16.957 ± 1.799 ^a^	6.42 ± 0.01 ^c^	6.16 ± 0.03 ^b^
***P. palmata***					
HW	95.0	8.736 ± 0.09 ^a^	9.724 ± 0.362 ^a^	6.46 ± 0.02 ^a^	6.44 ± 0.04 ^a^
PEF	46.3	8.571 ± 0.19 ^a^	9.214 ± 0.129 ^a^	6.39 ± 0.02 ^b^	6.52 ± 0.02 ^a^
PEF+HW	44.3/95	8.460 ± 0.17 ^a^	9.271 ± 0.037 ^a^	6.42 ± 0.03 ^a,b^	6.41 ± 0.07 ^a^
***U. lactuca***					
HW	95.0	6.213 ± 0.02 ^a^	6.740 ± 0.081 ^a^	6.07 ± 0.04 ^a^	6.25 ± 0.05 ^a^
PEF	51.0	6.006 ± 0.11 ^b^	6.261 ± 0.200 ^b^	5.94 ± 0.04 ^b^	5.33 ± 0.06 ^b^
PEF+HW	49.3/95	6.128 ± 0.01 ^a,b^	6.437 ± 0.094 ^a,b^	5.96 ± 0.03 ^b^	6.12 ± 0.05 ^c^

HW = hot water extraction; PEF = pulsed electric fields—assisted extraction; PEF + HW = combination of both techniques. For the PEF + HW, temperature is shown after the PEF and after the following hot water extraction procedure. Values are presented as mean ± SD (*n* = 3). Different lowercase letters indicate significant (*p* < 0.05) differences between treatments for each specie.

**Table 2:** Total phenolic content, total flavonoid content, and total carbohydrate content of seaweeds extracts (*A. esculenta*, *P. palmaria* and *U. lactuca*) produced by the investigated extraction methods.

Seaweed Specie and Extraction Method	Total Phenolic Content (µg GAE/g dw)	Total Flavonoid Content (µg QE/g dw)	Total Carbohydrate Content (mg GluE/g dw)
***A. esculenta***			
HW	8937.1 ± 785.7 ^a^	12232.8 ± 1248.7 ^a^	44.8 ± 1.5 ^a^
PEF	9368.2 ± 407.1 ^a^	12426.4 ± 848.3 ^a^	59.6 ± 1.1 ^b^
PEF+HW	8303.8 ± 594.1 ^a^	11636.8 ± 1393.6 ^a^	65.2 ± 2.7 ^c^
***P. palmata***			
HW	1850.5 ± 121.5 ^a^	805.0 ± 1.9 ^a^	510.5 ± 61.2 ^a^
PEF	1806.3 ± 104.2 ^a^	939.0 ± 95.9 ^a^	401.5 ± 43.8 ^a^
PEF+HW	1761.8 ± 97.8 ^a^	973.3 ± 45.8 ^a^	413.8 ± 26.5 ^a^
***U. lactuca***			
HW	1950.6 ± 109.5 ^a^	4533.1 ± 89.3 ^a^	249.5 ± 21.1 ^a^
PEF	1592.0 ± 95.8 ^b^	3427.3 ± 199.0 ^b^	106.3 ± 21.2 ^b^
PEF+HW	1709.4 ± 49.4 ^b^	4496.7 ± 589.4 ^a^	224.7 ± 19.1 ^a^

HW = hot water extraction; PEF = pulsed electric fields -assisted extraction; PEF + HW = combination of both techniques. Values are presented as mean ± SD (*n* = 3). Different lowercase letters indicate significant (*p* < 0.05) differences between treatments for each specie.

**Table 3:** Antioxidant capacities of seaweeds extracts (*A. esculenta*, *P. palmaria* and *U. lactuca*) produced by the investigated extraction methods: DPPH, ABTS and FRAP assays.

Seaweed Specie and Extraction Method	DPPH Scavenging Effect (%)	FRAP (µmol TE/100 g dw)	ABTS (µmol TE/100 g dw)
***A. esculenta***			
HW	93.8 ± 1.6 ^a^	984.4 ± 31.3 ^a^	86.5 ± 15.3 ^a^
PEF	91.8 ± 1.6 ^a^	960.7± 13.1 ^a^	89.2 ± 9.8 ^a^
PEF + HW	90.9 ± 1.0 ^a^	895.7 ± 46.8 ^a^	106.8 ± 8.3 ^a^
***P. palmata***			
HW	69.4 ± 7.3 ^a^	426.3 ± 65.5 ^a^	113.0 ± 5.6 ^a^
PEF	65.0 ± 7.2 ^a^	301.0 ± 7.9 ^a^	101.8 ± 1.5 ^a^
PEF + HW	56.4 ± 3.3 ^a^	302.7 ± 78.9 ^a^	97.4 ± 41.1 ^a^
***U. lactuca***			
HW	71.0 ± 5.7 ^a^	534.6 ± 42.4 ^a^	75.7 ± 12.5 ^a^
PEF	86.3 ± 0.5 ^b^	570.2 ± 26.5 ^a^	99.5 ± 5.9 ^a^
PEF + HW	71.9 ± 10.0 ^a^	547.8 ± 38.2 ^a^	81.6 ± 10.0 ^a^

HW = hot water extraction; PEF = pulsed electric fields—assisted extraction; PEF + HW = combination of both techniques. Values are presented as mean ± SD (*n* = 3). Different lowercase letters indicate significant (*p* < 0.05) differences between treatments for each specie.

**Table 4:** Collagenase, elastase, tyrosinase and hyaluronidase inhibitory activity of Icelandic seaweed extracts (*A. esculenta*, *P. palmaria* and *U. lactuca*) produced by the investigated extraction methods.

Samples	Collagenase Inhibition (%)	Elastase Inhibition (%)	Tyrosinase Inhibition (%))	Hyaluronidase Inhibition (%)
***A. esculenta***				
HW	71.7 ± 8.6 ^a^	73.4 ± 2.5 ^a^	95.5 ± 2.5 ^a^	100.0 ± 0.0 ^a^
PEF	90.8 ± 3.0 ^b^	73.8 ± 7.5 ^a^	92.9 ± 2.6 ^a^	100.0 ± 0.0 ^a^
PEF+HW	68.3 ± 5.6 ^a^	71.1 ± 3.0 ^a^	90.5 ± 4.6 ^a^	100.0 ± 0.0 ^a^
***P. palmata***				
HW	4.3 ± 7.4 ^a^	NI	NI	91.8 ± 0.5 ^a^
PEF	1.6 ± 1.8 ^a^	NI	NI	91.9 ± 0.0 ^a^
PEF + HW	2.3 ± 4.0 ^a^	NI	NI	89.5 ± 2.8 ^a^
***U. lactuca***				
HW	2.3 ± 2.1 ^a^	NI	NI	93.4 ± 0.6 ^a^
PEF	1.7 ± 2.9 ^a^	NI	NI	96.8 ± 0.4 ^b^
PEF + HW	1.5 ± 2.6 ^a^	NI	NI	97.3 ± 1.0 ^b^
Inhibitor from kit *	74.9 ± 1.2	NT	NT	NT
EGCG (5 mM) *	63.15	NT	NT	NT
EGCG (0.5 mM) *	13.70	NT	NT	NT
Quercetin (1mM) *	NT	100.0 ± 0.0	88.2 ± 1.4	NT
Quercetin (0.5 mM) *	NT	58.7 ± 11.7	74.8 ± 0.5	NT
Tannic acid (1 mM) *	NT	NT	NT	94.3 ± 0.2
Tannic acid (0.5 mM) *	NT	NT	NT	88.5 ± 3.2

HW = hot water extraction; PEF = pulsed electric fields -assisted extraction; PEF + HW = combination of both techniques. * Positive standards. No inhibition (NI). Not tested (NT). Epigallocatechin-3-gallate (EGCG). Values are presented as mean ± SD (*n* = 3). Different lowercase letters indicate significant (*p* < 0.05) differences between treatments for each specie.

**Table 5:** Pearson correlation coefficients between chemical components of the produced seaweed extracts, antioxidant capacity and inhibition effects on enzymes.

	Chemical Composition			Antioxidant Capacity			Enzyme Inhibition			
Variables	TPC	TFC	TCC	DPPH	FRAP	ABTS	Collag	Elast	Tyros	Hyalur
**TPC**	1	0.95 ***	−0.70 ***	0.74 ***	0.91 ***	−0.04	0.98 ***	0.99 ***	0.99 ***	0.42 *
**TFC**		1	−0.84 ***	0.82 ***	0.97 ***	−0.24	0.93 ***	0.94 ***	0.95 ***	0.54 **
**TCC**			1	−0.91 ***	−0.86 ***	0.31	−0.68 ***	−0.71 ***	−0.71 ***	−0.60 **
**DPPH**				1	0.88 ***	−0.19	0.74 ***	0.75 ***	0.75 ***	0.52 **
**FRAP**					1	−0.25	0.90 ***	0.91 ***	0.91 ***	0.61 **
**ABTS**						1	−0.03	0.00	0.00	−0.18
**Collag**							1	0.98 ***	0.98 ***	0.42 *
**Elast**								1	0.998 ***	0.43 *
**Tyros**									1	0.43 *

* *p* ≤ 0.05, ** *p* ≤ 0.01, *** *p* ≤ 0.001.

**Table 6:** Experimental design for bioactives extraction from *A. esculenta*, *P. palmata* and *U. lactuca*.

Seaweed Species	Extraction Procedure		
*A. esculenta*	HW (95 °C, 45 min) *n* = 3	PEF (10 min) *n* = 3	PEF + HW (10 min + 95 °C, 45 min) *n* = 3
*P. palmata*	HW (95 °C, 45 min) *n* = 3	PEF (10 min) *n* = 3	PEF + HW (10 min + 95 °C, 45 min) *n* = 3
*U. lactuca*	HW (95 °C, 45 min) *n* = 3	PEF (10 min) *n* = 3	PEF + HW (10 min + 95 °C, 45 min) *n* = 3

HW = hot water extraction; PEF = pulsed electric fields -assisted extraction; PEF + HW = combination of both techniques.
